# Effect of weight loss before in vitro fertilization in women with obesity or overweight and infertility: a systematic review and meta-analysis

**DOI:** 10.1038/s41598-024-56818-4

**Published:** 2024-03-14

**Authors:** Hye Gyeong Jeong, Sumin Cho, Ki-Jin Ryu, Tak Kim, Hyuntae Park

**Affiliations:** 1 Department of Obstetrics and Gynecology, Korea University Anam Hospital, Korea University College of Medicine, Seoul, Korea; 2 Department of Obstetrics and Gynecology, Seoul National University College of Medicine, Seoul, Korea

**Keywords:** Obese, Overweight, Weight loss, In vitro fertilization, Fertility, Endocrinology, Medical research, Risk factors

## Abstract

The effect of weight loss before in vitro fertilization (IVF) procedures on pregnancy outcomes in women with overweight or obesity and infertility remains controversial. In this systematic review and meta-analysis, we investigated whether weight loss before IVF in these women affected the IVF results and reproductive outcomes. PubMed, Embase, and the Cochrane Library databases were searched from the inception dates until December 2022, using combinations of relevant keywords. Only six randomized controlled trials, including 1627 women with obesity or overweight, were analyzed. The weight change in the intensive care group, compared to the control group who underwent IVF without weight loss was – 4.62 kg (mean difference; 95% confidence interval [CI] − 8.10, − 1.14). Weight loss before IVF did not significantly increase the live birth rate in women with obesity or overweight and infertility (odds ratio, 1.38; 95% CI 0.88, 2.10). The clinical pregnancy, miscarriage, ongoing pregnancy, and ectopic pregnancy rates did not differ between the weight loss and control groups before IVF. This meta-analysis demonstrated that even significant weight loss before IVF in women with obesity or overweight and infertility did not improve the live birth, clinical pregnancy, ongoing pregnancy, or ectopic pregnancy rates. *PROSPERO Registration Number:* CRD42023455800.

## Introduction

Obesity is no longer a local or national problem but a global health problem. The prevalence of obesity and overweight is rapidly increasing worldwide^1^. Over the past few decades, obesity has surged, contributing to numerous health concerns such as fertility issues^2^. Although the association of obesity with diabetes, hypertension, metabolic syndrome, cardiovascular disease, and other serious health problems, is well known, the effects of being obese or overweight on reproductive health remain controversial.

Infertility is defined as a condition in which pregnancy does not occur even after unprotected regular sexual intercourse for more than 1 year^3^. The causes of infertility are categorized into several factors such as age, ovulation-related issues, uterine-related concerns, male factors, and unexplained factors. Obesity could be a cause of infertility in relation to ovulation- and uterine-related factors, particularly endometrial factors, and is known to affect menstruation, natural fertility and fecundity rates, and the success rates and the safety of infertility treatment^4,5^. Additionally, obesity is associated with maternal and fetal health problems and increases the incidence of complications during pregnancy. Epidemiological evidence has shown strong associations of obesity with infertility^6^, miscarriage and pregnancy loss^7–9^, as well as increased fetal and maternal complications during pregnancy^10–12^. Consequently, experts, major medical societies, and public health programs have endorsed or even mandated the reduction of body weight before infertility treatment in women with obesity^13,14^. Women with overweight or obesity and infertility are advised to reduce their body weight before pregnancy and receive infertility treatment to improve reproductive and fertility outcomes, although corroborative evidence from previous studies has been lacking and inconsistent^15^.

Various lines of evidence and different studies have demonstrated that obesity itself affects reproductive and pregnancy outcomes, but it remains unclear whether losing weight in patients with obesity and infertility affects artificial reproductive therapy (ART) results. Some studies have also reported that women with obesity have a reduced response to ovarian hyperstimulation during ART, which affects egg quality and endometrial function and increases the miscarriage rate^16–21^. Obesity may negatively impact fertility, but ART could potentially mitigate decreased fertility. Few studies have assessed the quality of evidence regarding whether the reproductive or pregnancy outcomes improve if women with obesity or overweight lose weight, as compared to those who do not lose weight, before performing ART, such as in vitro fertilization (IVF).

This study aimed to evaluate the effect of intensive weight loss immediately before IVF on weight loss and reproductive outcome improvement in obese and overweight women with infertility through a meta-analysis and systematic review of previously published studies.

## Results

**Figure 1:**
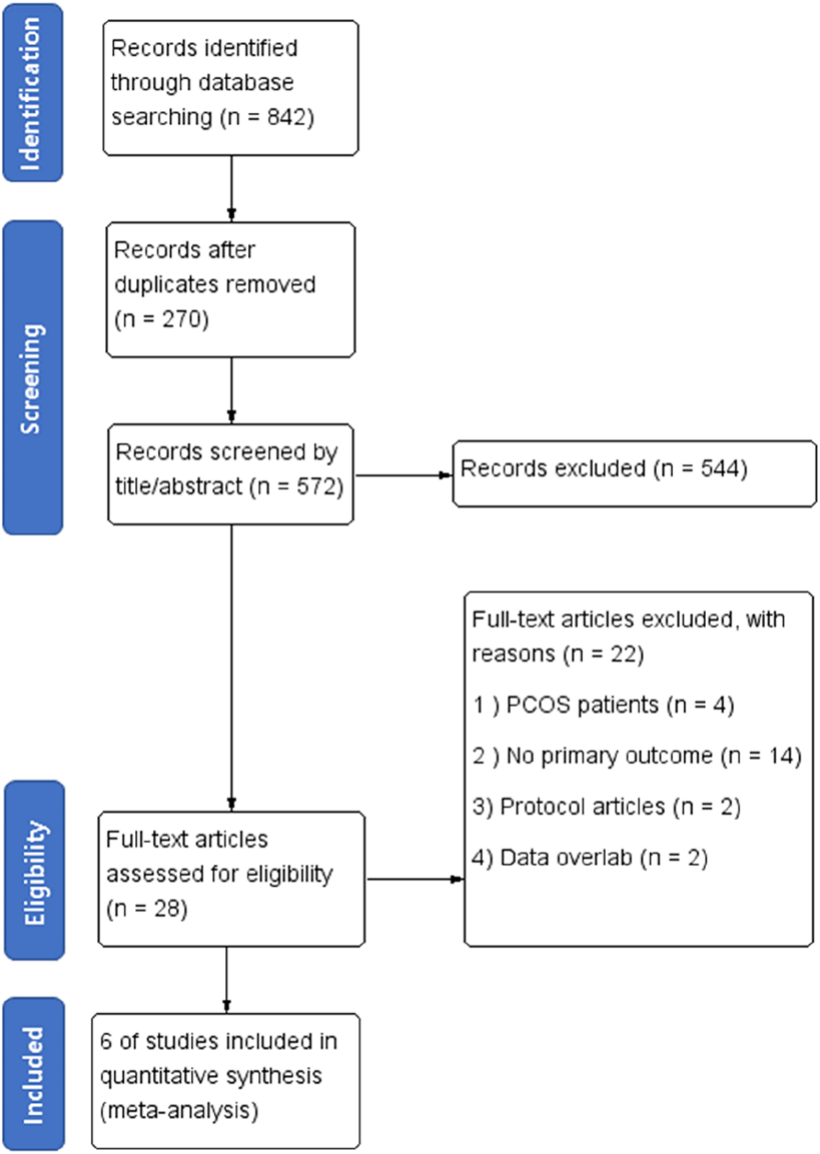
Flowchart of study selection for systematic review and meta-analysis.

**Figure 2:**
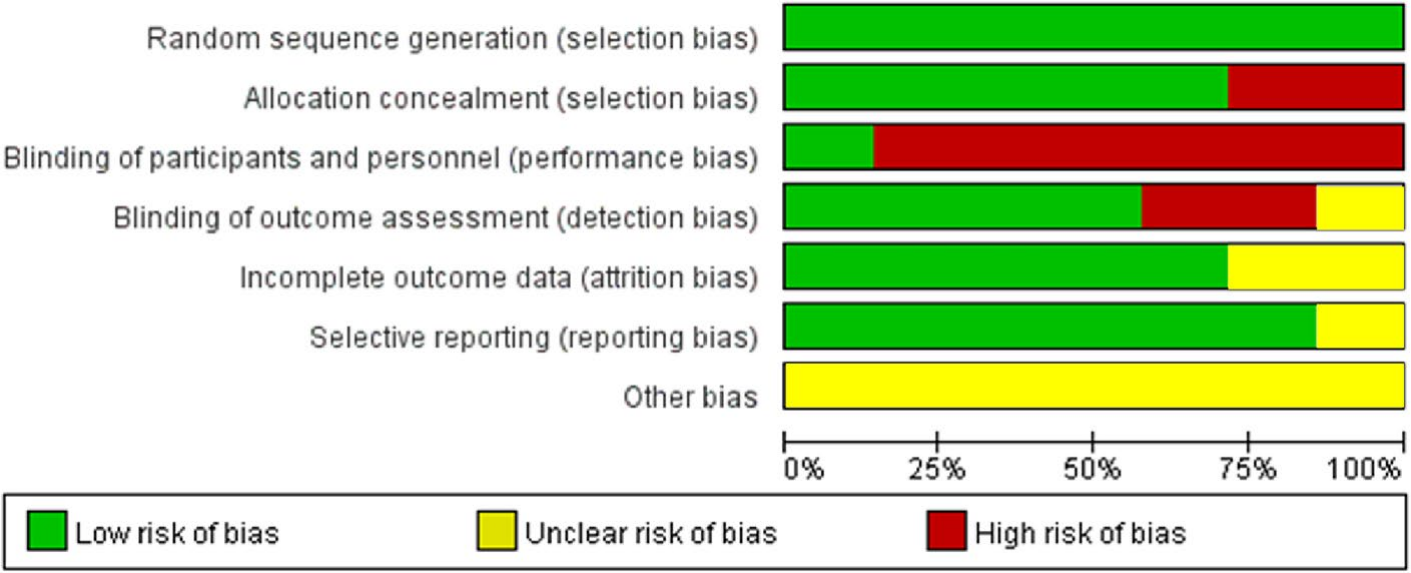
Risk of bias summary.

The search strategy yielded 842 articles, all of which were obtained from electronic databases (Fig. 1). After removing duplicate articles, the authors excluded 544 publications that did not fulfill the selection criteria after screening the title or abstract. Of the remaining 28 articles, 22 were excluded because they were studies on patients with polycystic ovary syndrome (PCOS), had a different primary outcome than the one selected, were protocol articles, or had overlapping data. For the remaining six articles, we obtained the full text for a detailed investigation and extracted the data necessary for analysis. The summarized characteristics of the included studies are displayed in Table 1^22–27^. A summary of the risk of bias is presented in Fig. 2.

**Table 1:** Characteristics of the studies included in the meta-analysis.

Investigator (year), country	Study design	Inclusion criteria	Sample size	Main outcomes eligible for meta-analysis	Adjusted for the following confounding factors
Moran (2011), Australia^22^	RCT	18–40 years, 28 ≤ BMI < 45 kg/m^2^, GnRH agonist protocol	Lifestyle treatment (diet & exercise) (n = 18), control (n = 20)	Live birth rates Weight change Clinical pregnancy rate	Unadjusted
Sim (2014), Australia^23^	RCT	18–37 years, BMI ≥ 30 kg/m^2^, intending to commence IVF, ICSI, or cryostored embryo transfer treatment at RPAH Fertility Unit	Intensive dietary support & activity intervention (n = 27), Allocated to standard care (n = 17)	Live birth rates Weight change Clinical pregnancy rate Miscarriage rate (< 6 weeks, 6–12 weeks)	Number of fertility treatment cycles
Mutsaerts (2016), Netherlands^24^	RCT	Subfertile women, 18–39 years, 29 ≤ BMI < 40 kg/m^2^	6 months’ lifestyle-intervention program (n = 280), control (n = 284)	Live birth rates Weight change Clinical pregnancy rate Ongoing pregnancy rate Multiple pregnancy rate Ectopic pregnancy rate	Unadjusted
Einarsson (2017), Sweden^25^	RCT	< 38 years, planning IVF, 30 ≤ BMI < 35 kg/m^2^	Weight reduction group (n = 152), control (n = 153)	Live birth rates Weight change Clinical pregnancy rate Ectopic pregnancy rate	Unadjusted
Espinós (2017), Spain^26^	RCT	18–37 years, 30 < BMI < 40 kg/m^2^, presenting for their first IVF cycle	12-week diet and exercise intervention (n = 21), control (n = 20)	Live birth rates Weight change Multiple pregnancy rate Miscarriage rate	Unadjusted
Wang (2021), China^27^	RCT	20–40 years, 25 kg/m^2^ ≤ BMI, scheduled for IVF or ICSI	Orlistat (n = 439), placebo (n = 438)	Live birth rate Weight change Clinical pregnancy rate Ongoing pregnancy rate Miscarriage rate Ectopic pregnancy rate	Unadjusted

RCT, randomized controlled trial; BMI, body mass index; GnRH, gonadotropin-releasing hormone; IVF, in vitro fertilization; ICSI, intracytoplasmic sperm injection.

### Study characteristics

All studies included in this systematic review and meta-analysis were randomized controlled trials (RCTs). The definitions of obesity and overweight differed slightly between studies but mainly included patients with a body mass index (BMI) ≥ 25 kg/m^2^, who were undergoing or planning to undergo IVF due to infertility. In two studies, weight loss was achieved using medications, whereas in the other five studies, it was achieved through lifestyle modifications, such as exercise or diet.

### Quality of studies

The quality of the included studies was heterogenous. All included studies were RCTs, so the overall possibility of bias was low. However, due to the nature of the intervention, most studies showed high risk in terms of blinding of participants to treatment allocation. The summary of the risk of bias is shown in Fig. 2.

### Primary outcome

#### Weight changes

**Figure 3:**
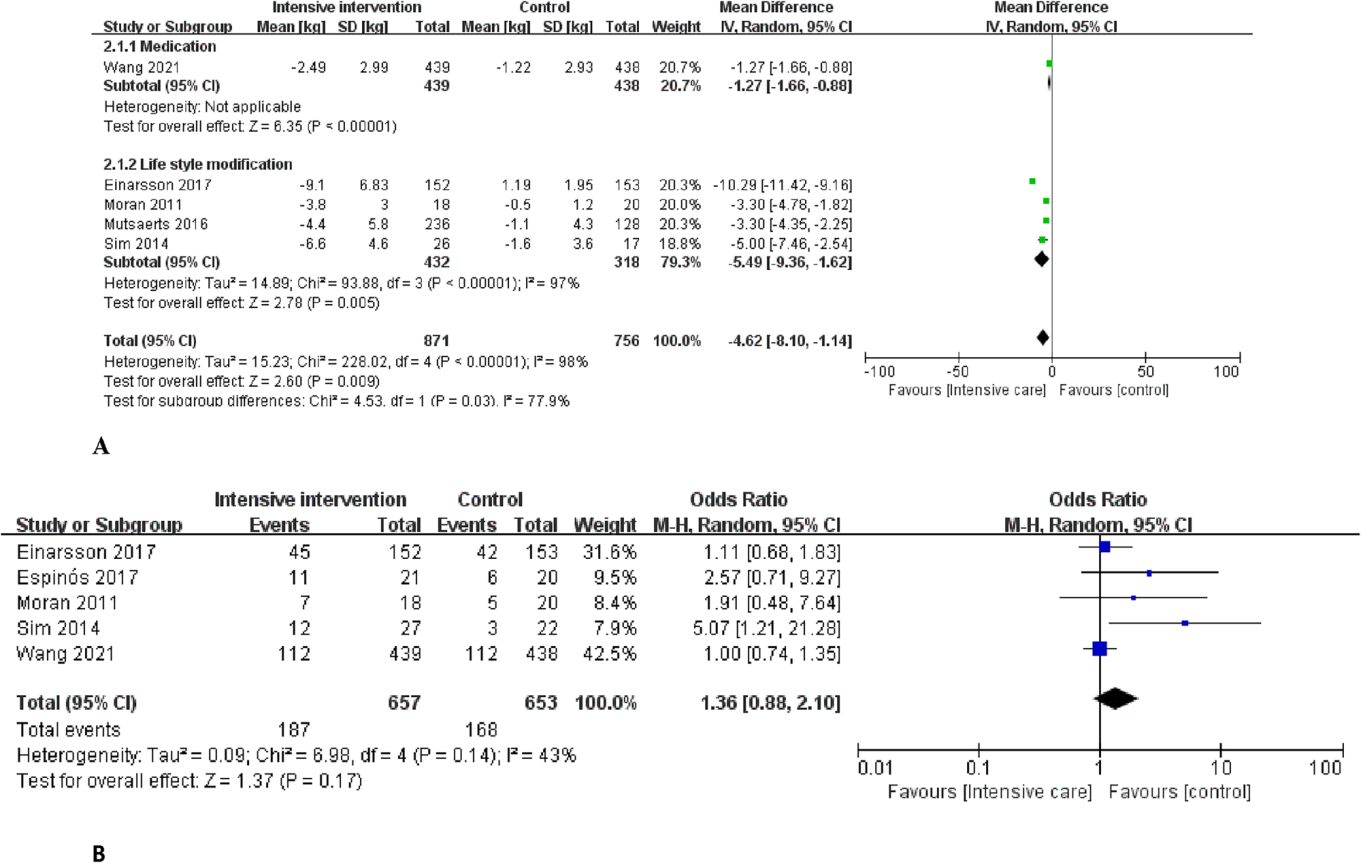
Primary outcomes: (**A**) Weight change in women who underwent intensive intervention for weight loss prior to in vitro fertilization. (**B**) The live birth rate in all women who lost weight prior to in vitro fertilization. (**C**) The live birth rate in women who lost weight with the use of medication prior to in vitro fertilization.

Six studies including 1627 women with infertility who were obese or overweight were included. The weight change in the intensive intervention group using medication or lifestyle modification compared with the control group that underwent IVF without weight loss was found to be − 4.62 kg [mean difference [MD]; 95% confidence intervals [CI] − 8.10, − 1.14). One study on weight loss using medication was included^27^. Compared with the control group, the weight change in the intensive intervention group using medication was − 2.49 kg (MD; 95% CI − 1.66, − 0.88). Four studies on weight loss through lifestyle modification were included. Compared with the control group, the weight change in the intensive intervention group was − 5.49 kg (MD; 95% CI − 9.36, − 1.62; Fig. 3A). The results were significant. Significant heterogeneity was also observed among the included studies (I^2^ = 98%; *p* < 0.00001).

#### Live birth rates

Weight reduction before IVF did not significantly improve the live birth rate in obese or overweight women with infertility (odds ratio [OR], 1.36; 95% CI 0.88, 2.10; Fig. 3B). There was significant heterogeneity among the included studies (I2 = 43%; *p* = 0.14). Even when weight loss was achieved through lifestyle modification and medication, a significant increase in the live birth rate in the intensive intervention group compared with the control group could not be confirmed.

### Secondary outcomes

#### Clinical pregnancy rate

**Figure 4:**
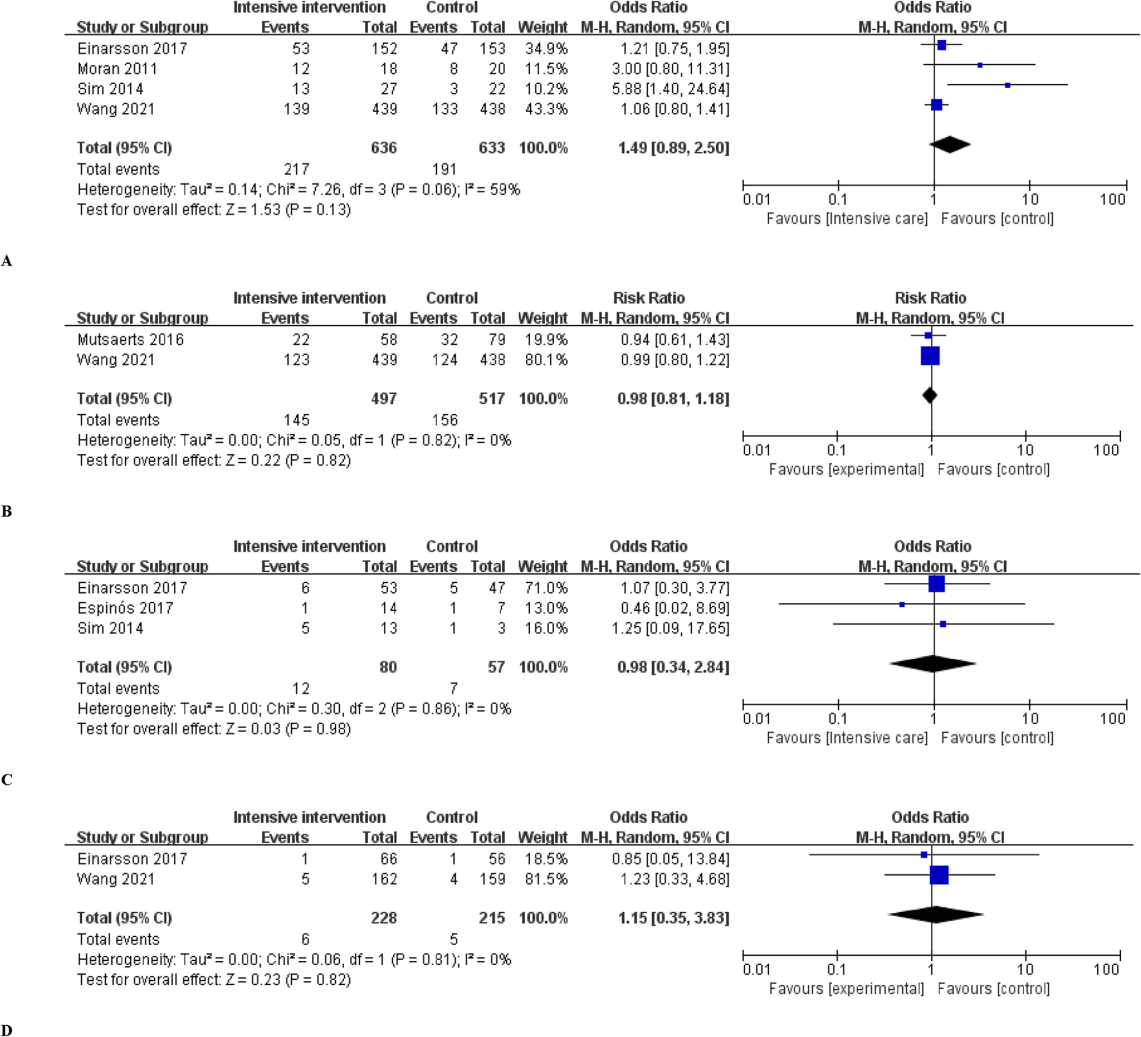
Secondary outcomes: (**A**) Clinical pregnancy rate. (**B**) Ongoing pregnancy rate. (**C**) Miscarriage rate. (**D**) Ectopic pregnancy rate in women with obesity or overweight who lost weight prior to in vitro fertilization.

For the clinical pregnancy rate, data collected from four studies^22,23,25,27^ were analyzed. Clinical pregnancy was defined as a case in which a G-sac was confirmed using ultrasonography. The clinical pregnancy rate was not significantly improved in the intensive intervention group compared with the control group [OR, 1.49; 95% CI 0.89, 2.50] (Fig. 4A). There was significant heterogeneity among the included studies (I2 = 59%; *p* = 0.06).

#### Ongoing pregnancy rate

Pooled data analysis from two studies^24,27^ showed that the ongoing pregnancy rate was not significantly higher in the intensive intervention group compared with control group before IVF (OR, 0.98; 95% CI 0.81, 1.18] (Fig. 4B). No significant heterogeneity was observed in the included studies (I2 = 0%).

#### Miscarriage rate

Pooled data analysis from three studies^23,25,26^ showed that the miscarriage rate was not significantly higher in the intensive intervention group compared with control group before IVF (OR, 0.98; 95% CI 0.34, 2.84) (Fig. 4C). No significant heterogeneity was observed in the included studies (I2 = 0%).

#### Ectopic pregnancy rate

According to the pooled data analysis of two studies^25,27^, the ectopic pregnancy rate was not significantly different between the intensive intervention and control groups (OR, 1.15; 95% CI 0.35, 3.83; Fig. 4D). No significant heterogeneity was observed in the included studies (I2 = 0%).

## Discussion

No previous systematic review and meta-analysis has reported on whether weight loss immediately before ART, such as IVF or intracytoplasmic sperm injection (ICSI), improves reproductive and pregnancy outcomes in women with obesity or overweight and infertility, compared to outcomes in a control group that did not undergo intensive weight loss. Obesity has become a global epidemic^5,28^. An increasing number of women with overweight or obesity are turning to ART to address fertility concerns. Thus, the impact of overweight or obesity on the reproductive outcomes of IVF or ICSI treatments is of interest to reproductive specialists and patients.

Previous studies have investigated how being obese or overweight before IVF affects reproductive outcomes; however, their results have been inconsistent. In a systematic review and meta-analysis, Sermondade et al. reported that obesity in women before IVF had a negative effect on the live birth rate^29^. A meta-analysis of 21 studies reported that the live birth rate after IVF decreased by a risk ratio of 0.85 (95% CI 0.82, 0.87) in women with obesity with a BMI ≥ 30 kg/m^2^ as compared to the rate in normal-weight women^29^. Some RCTs reported that weight loss before IVF increased the live birth rate; however, the sample size in these trials was small^23^. Furthermore, other RCTs highlighted a decreased live birth rate; therefore, the results of these studies were inconsistent^24^.

According to a committee opinion published by the American Society for Reproductive Medicine (ASRM) in 2021, obesity lowers the birth rate after IVF by causing ovulation disorders, reduced ovarian responsiveness to ovulation-inducing drugs, and functional changes in the endometrium^5^. In addition, maternal obesity during pregnancy has been reported to have a negative impact on maternal and fetal complications, such as gestational diabetes, gestational hypertension, preeclampsia, cesarean section rate, stillbirth, macrosomia, congenital malformations, and neonatal intensive care unit admission^5^.

Various methods to manage obesity have been proposed, and this meta-analysis demonstrated that lifestyle modification and medication are both effective in achieving weight loss. Previous studies have suggested that phentermine, diethylpropion, benzphetamine, phendimetrazine, orlistat, naltrexone SR/bupropion SR, liraglutide, and phentermine/topiramate ER are effective medications for weight loss, and demonstrated that weight loss was effective when the medications were used according to their purpose^30,31^. However, side effects and contraindications should be carefully considered. In this meta-analysis, only orlistat was included as a weight-loss medication, as only RCTs were included in the analysis. Although bariatric surgery is an important method to aid in weight reduction along with lifestyle modifications and medication, it is recommended that such women delay pregnancy for 1 year after surgery, to avoid fetal nutritional deficiencies^32,33^. None of the included studies attempted weight loss through bariatric surgery.

In anovulatory women with obesity, particularly those with PCOS, weight loss increases the chances of ovulation and pregnancy without assistance. In anovulatory women with obesity, weight loss improves the ovulation rate in response to ovulation induction^34,35^. However, these studies did not find any improvement in live birth rates^36^.

The live birth rate, which was the primary outcome of this study, did not differ significantly between the intensive intervention and control groups. Previous studies have not demonstrated that weight reduction before pregnancy improves live birth rates after non-ART or IVF in women with obesity with normal ovulation^5^. Although weight reduction before IVF may reduce the complications of IVF procedures, the effect of weight reduction before pregnancy on maternal and fetal complications remains unclear.

In some countries or fertility centers, infertility treatment is allowed only when the patient’s BMI is below a certain threshold; for patients with BMIs above this threshold, infertility treatment is not allowed until the patient reduces their weight^37,38^. The Royal Australian and New Zealand College of Obstetrics and Gynecology policy on assisted reproduction has established that a BMI ≥ 35 kg/m^2^ is a known risk factor for pregnancy and delivery and is an absolute contraindication to ART^39^. In addition, in the UK, postponing treatment such as IVF is recommended until the BMI is < 35 kg/m^2^ or within the normal range, especially if the patients’ BMI is ≥ 35 kg/m^2^^2,40^ However, based on medical evidence, providing a definitive globally agreed-upon BMI threshold for infertility treatment is difficult. In contrast, considerable evidence against the policy of determining fertility treatment based on the BMI threshold exists, because obesity or being overweight does not significantly affect the outcome of infertility treatments, such as IVF. Furthermore, women with obesity along with infertility can safely undergo IVF procedures^41^. Thus, the ASRM committee suggests that obesity should not be the only reason for denying access to infertility treatments to patients or couples^5^. The results of this meta-analysis also support these suggestions, as weight reduction immediately before IVF in women with obesity or overweight with infertility does not increase the live birth rate.

This meta-analysis compared pregnancy and reproductive outcomes between women with obese or overweight and who lost weight immediately before IVF and those who did not lose weight. This analysis differs from previous studies that compared pregnancy outcomes in women with normal weight and outcomes in those with obesity or overweight and infertility. The strength of this study is that a meta-analysis including only RCTs was conducted to secure the evidence for this study. In addition, a relatively large number of patients were analyzed, including relatively recent large-scale studies. In addition, as the analysis was conducted on studies on weight loss immediately before IVF, additional reproductive and obstetric results were obtained. However, the quality of evidence may be limited by the heterogeneity among studies and the risk of bias.

In conclusion, this meta-analysis suggested that intensive weight loss in women with obesity or overweight and infertility immediately before IVF was effective in reducing body weight, but did not improve pregnancy outcomes, such as live birth, clinical pregnancy, ongoing pregnancy, miscarriage, and ectopic pregnancy rates. This suggests that intensive weight loss immediately before IVF in women with obesity or overweight and infertility did not improve reproductive outcomes.

## Methods

The Preferred Reporting Items for Systematic Reviews and Meta-Analyses checklist was used for this review.

### Criteria for considering studies for systematic review and meta-analysis

In this meta-analysis, only RCTs were considered, and most clinical trial participants included patients with obesity or overweight according to World Health Organization standards. Although some studies used different BMI criteria for the participants, the BMI in all studies corresponded to the criteria for overweight or obesity. The term “intensive intervention” used in this analysis included the use of one or more drugs or exercise for weight loss, regardless of whether diet was included or not. Because all included studies were RCTs, approaches to weight loss varied across studies, including primarily a low-calorie diet followed by exercise therapy or other additional interventions aimed at weight loss. The control group also basically used weight loss methods such as a low-calorie diet. Therefore, the term “intensive” was used in groups where more than one method was used. Selecting only RCTs involving weight loss using only one method (either medication or exercise) would have reduced the number of studies included in the analysis. Additionally, including both methods allowed for the possibility of conducting subgroup analyses based on the type of intervention.

In the case of patients with PCOS-related infertility who were overweight or obese, weight loss could improve ovulation disorders and reproductive outcomes. Once the ovulation disorders were corrected, the need for ART decreased. Therefore, to exclude bias in the effect due to improvement in PCOS-related ovulation disorder and to examine only the effect of weight loss immediately before IVF on reproductive outcomes, studies that included only patients with PCOS were excluded from this meta-analysis.

### Search strategy

A literature search was conducted in PubMed, Embase, and the Cochrane Library on January 16, 2023. A combination of medical subject headings (MeSH) and text words were used: “overweight,” “obesity,” “female,” “fertilization in vitro,” “IVF (in vitro fertilization),” “ICSI (intracytoplasmic sperm injection),” “ART (Assisted reproductive technology)”, “weight loss”, “weight reduction”, “weight control”, “diet”, “exercise”, and “physical activity”.

Database searches were conducted using a combination of the following search terms: (“overweight” [MeSH Terms] OR “overweight” [Title/Abstract] OR “obese” [Title/Abstract] OR “obesity” [MeSH Terms] OR “obesity” [Title/Abstract] AND (“live birth rate” [MeSH Terms] OR “live birth rate” [Title/Abstract]) AND (“fertilization in vitro” [Title/Abstract] OR “in vitro fertilization” [Title/Abstract] OR “fertilization in vitro” [MeSH Terms] OR “fertilization in vitro” [Title/Abstract] OR “ivf” [Title/Abstract] OR “in vitro fertilization” [Title/Abstract] OR “sperm injections, intracytoplasmic” [MeSH Terms] OR “sperm injections, intracytoplasmic” [Title/Abstract] OR “intracytoplasmic sperm injections” [Title/Abstract] OR “icsi” [Title/Abstract]) AND (“weight loss” [MeSH Terms] OR “weight loss” [Title/Abstract]) OR (“weight reduction” [MeSH Terms] OR “weight reduction” [Title/Abstract]) OR (“weight management” [MeSH Terms] OR “weight management” [Title/Abstract]) OR (“diet” [MeSH Terms] OR “diet” [Title/Abstract]) OR (“exercise” [MeSH Terms] OR “exercise” [Title/Abstract]) OR (“physical activity” [MeSH Terms] OR “physical activity” [Title/Abstract]) AND (English [lang] AND (“humans” [MeSH Terms] OR (“women” [MeSH Terms] OR “women” [All Fields] OR “woman” [All Fields])).

The literature was limited to only the studies published in English. Animal experiments were excluded, and only RCTs involving humans were included in this analysis. The searches were performed independently by two researchers, HGJ and HTP.

### Study selection

Two authors (HT and HG) independently screened the titles and abstracts of all searched papers and excluded the citations that were deemed irrelevant. Three authors (SM, KJ, and T) retrieved full texts of potentially relevant articles and evaluated them for inclusion according to predetermined criteria. Methodological quality assessment was performed using the Cochrane Handbook.

### Excluded studies

This systematic review and meta-analysis did not include data from clinical studies or conference abstracts. Non-RCTs, such as retrospective or cohort studies, were also excluded. Additionally, articles that did not evaluate the selected primary outcome were also excluded.

### Data extraction

Data extraction from the included articles was conducted independently by two authors (HG and HT). To characterize the included studies, the following details were extracted: study author, year of publication, study period, country, study design, eligibility criteria, participants’ BMI, weight loss method (exercise, diet, or medication), method of fertilization, type of embryo transfer, method of conception, and outcome of IVF. The sample size, participant age, reproductive outcomes, and live birth rate were recorded for each group (intensive weight loss before IVF vs. control).

### Outcome measure

The primary outcomes assessed were weight change and live birth rate, and the secondary outcomes included other reproductive measures, such as clinical pregnancy, ongoing pregnancy, miscarriage, and ectopic pregnancy rates after the intervention in the intensive intervention and control groups. A subgroup analysis of birth rate was performed by stratifying the weight control group into two subgroups: one using medication and the other employing lifestyle modifications for weight loss. Our definition of live birth was the delivery of a live fetus(es), regardless of gestational age, referring to the definitions of the articles included. The clinical pregnancy rate used in this analysis was defined as cases where a gestational sac was confirmed on ultrasound. Ongoing pregnancy was defined as a case where a viable pregnancy was maintained beyond 10 weeks of gestation. Miscarriage was defined as the case where clinical pregnancy was terminated after 6 weeks of gestation. Ectopic pregnancy was defined as pregnancy occurring outside the uterine cavity. Although some studies did not precisely adhere to this definition, most could be classified according to these definitions; thus, these differences were within an acceptable range.

### Bias assessment

The risk of bias was independently evaluated by two authors (HG and HT) using the Cochrane Risk of bias tool^42^, considering the categories of high, low, and unclear depending on the level of bias. We included only RCTs, to reduce the risk of bias.

### Statistical analysis

The meta-analysis results were combined and analyzed using Review Manager (RevMan) version 5.4.1 (The Nordic Cochrane Centre, The Cochrane Collaboration, Copenhagen, Denmark). We pooled the ORs reported in each included study, with 95% CIs as the measure of the association between weight loss before IVF and reproductive results, such as live birth, clinical pregnancy, and miscarriage rates. Continuous variables were presented as mean differences, and the accuracy of the estimates was evaluated with 95% CIs. A random-effects model was used to account for the differences in actual effects.

## Data Availability

The datasets used and analyzed during the current study are available from the corresponding author upon reasonable request.
